# Supplementary figures and images for: Mitochondrial dysfunction in rheumatoid arthritis: A comprehensive analysis by integrating gene expression, protein-protein interactions and gene ontology data
Source: PLoS One. 2019 Nov 8;14(11):e0224632. doi: 10.1371/journal.pone.0224632 (PMC6839853; doi:10.1371/journal.pone.0224632)

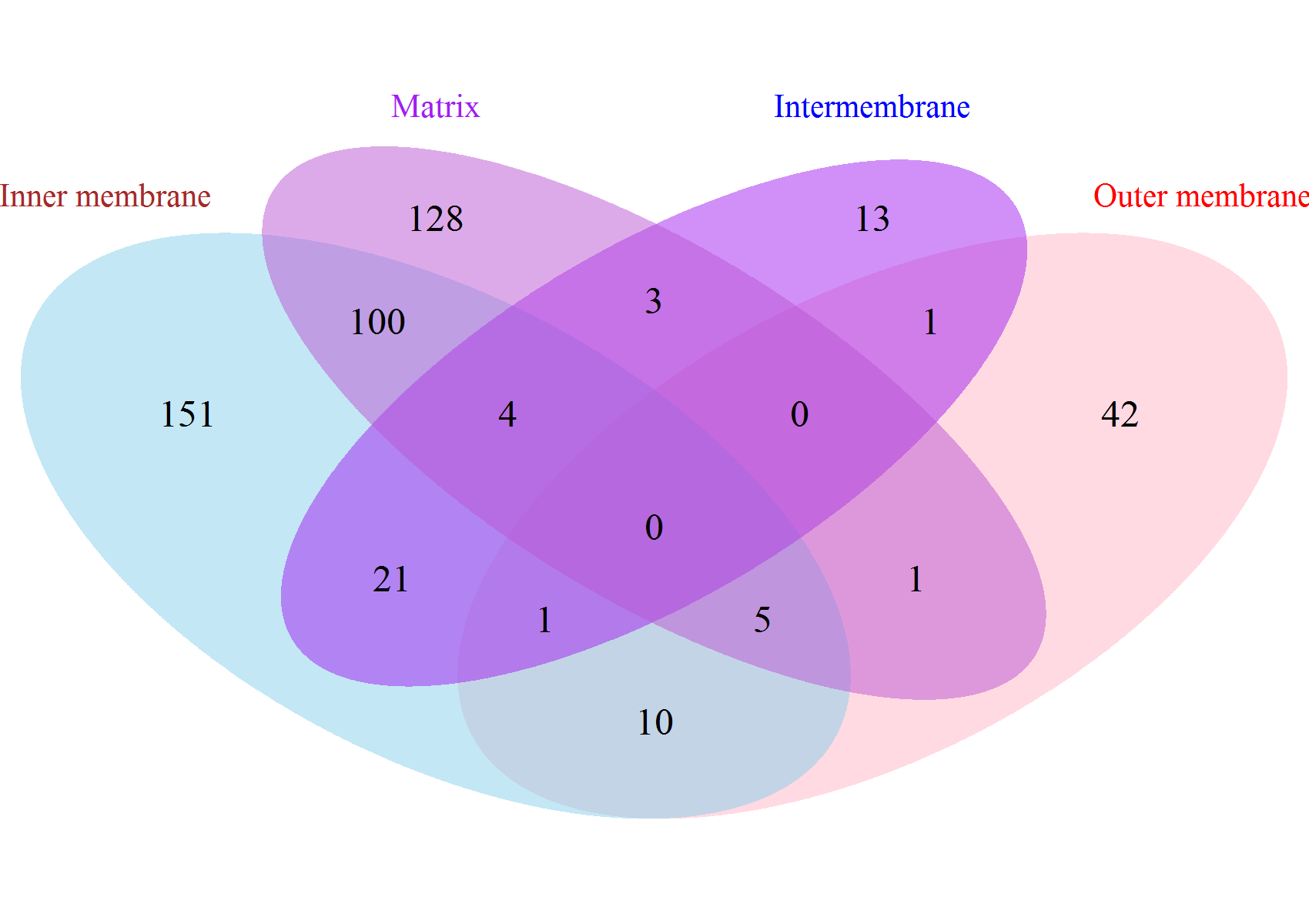

Supplement: S1 Fig — (TIF) [file pone.0224632.s002.tif]

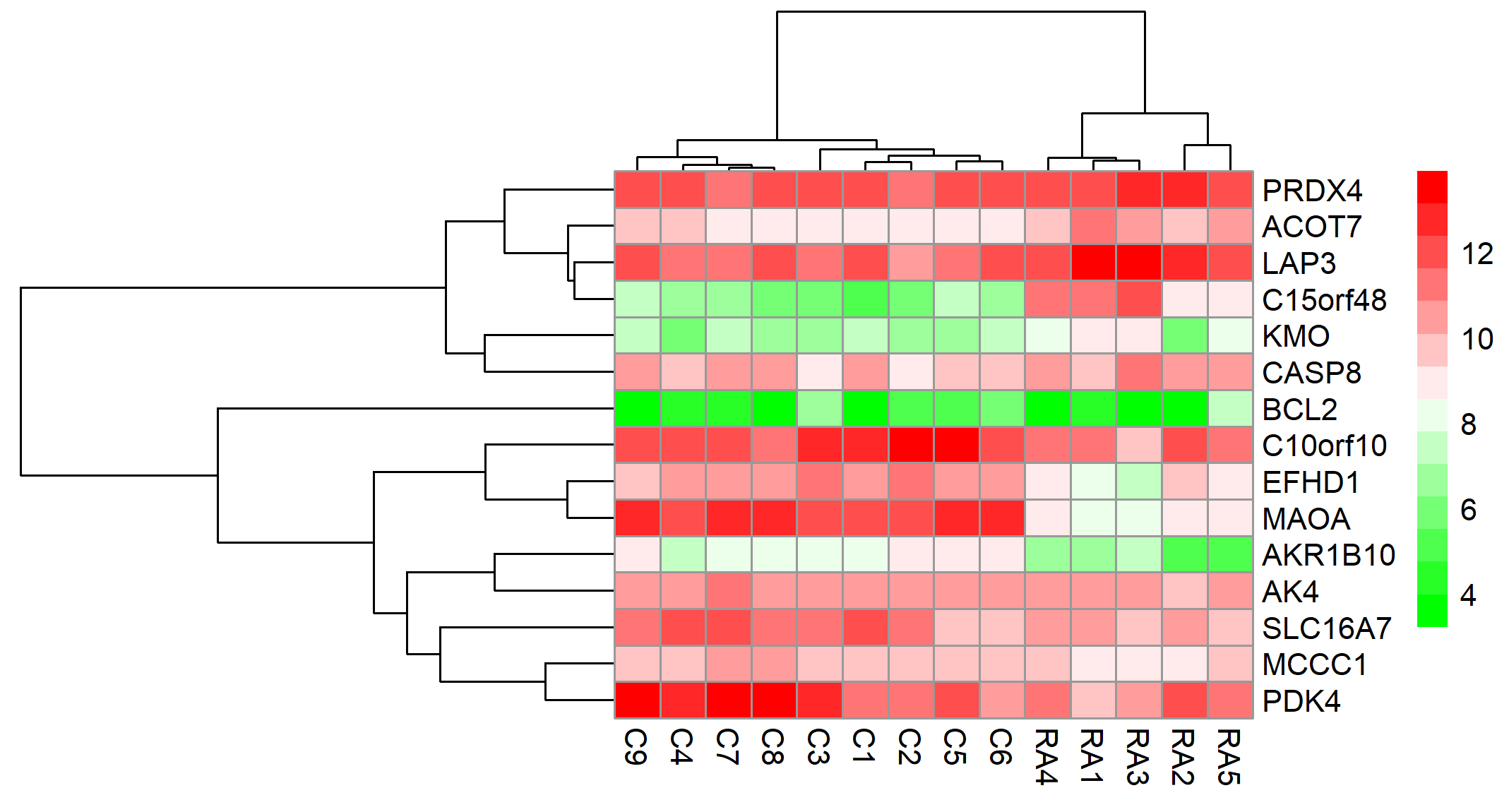

Supplement: S2 Fig — (TIF) [file pone.0224632.s003.tif]

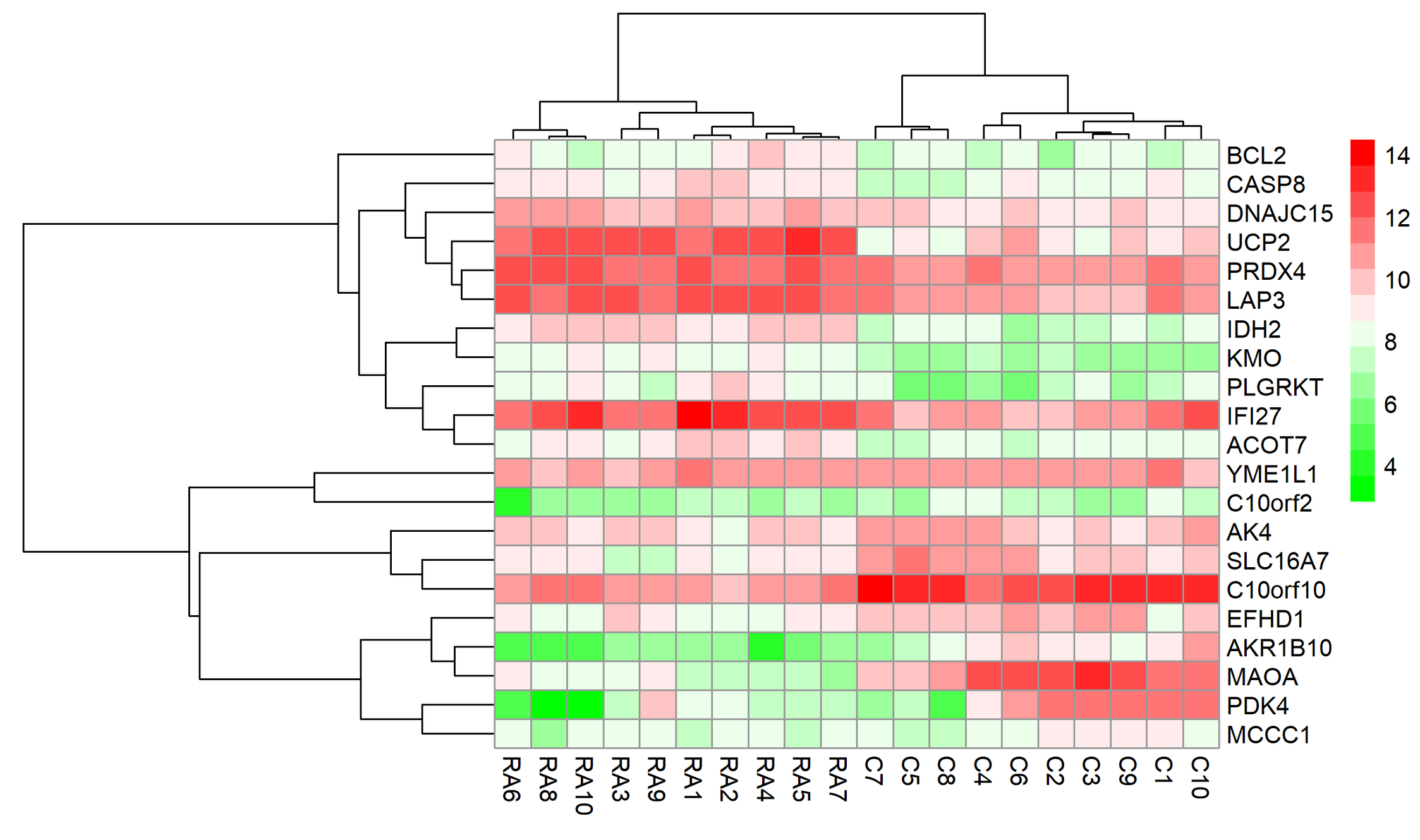

Supplement: S3 Fig — (TIF) [file pone.0224632.s004.tif]

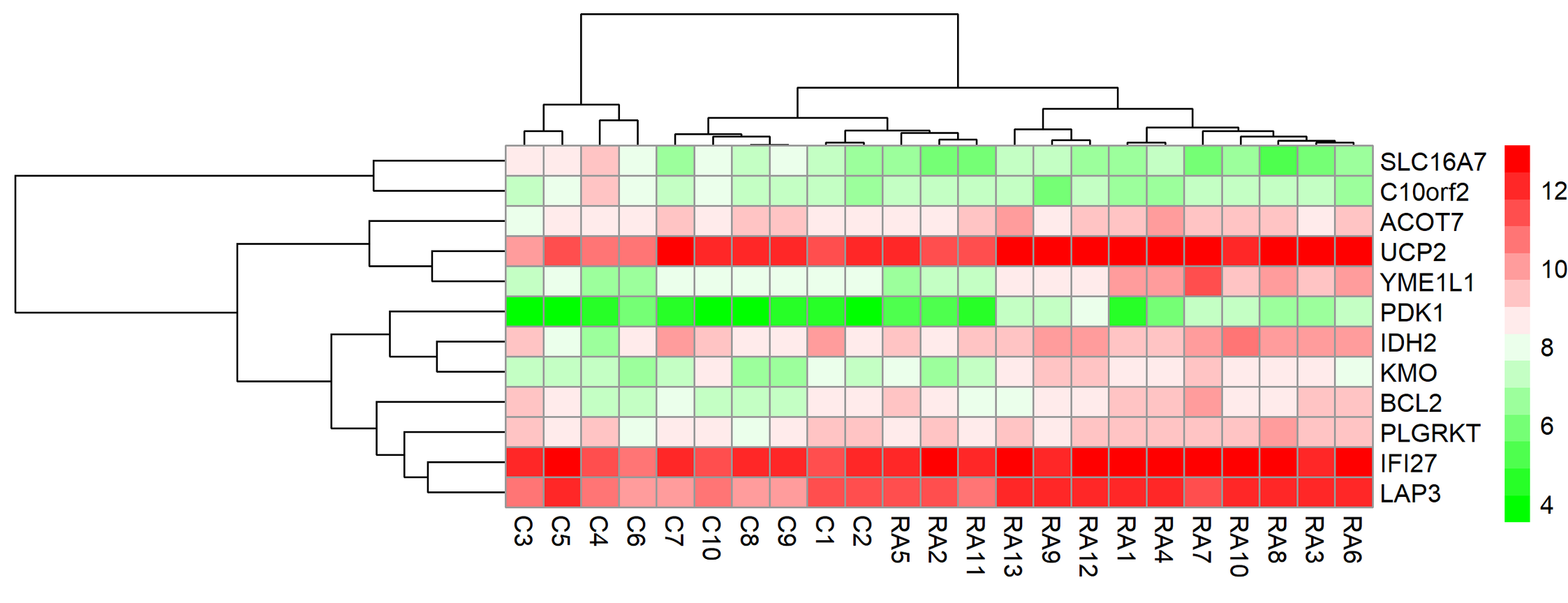

Supplement: S4 Fig — (TIF) [file pone.0224632.s005.tif]

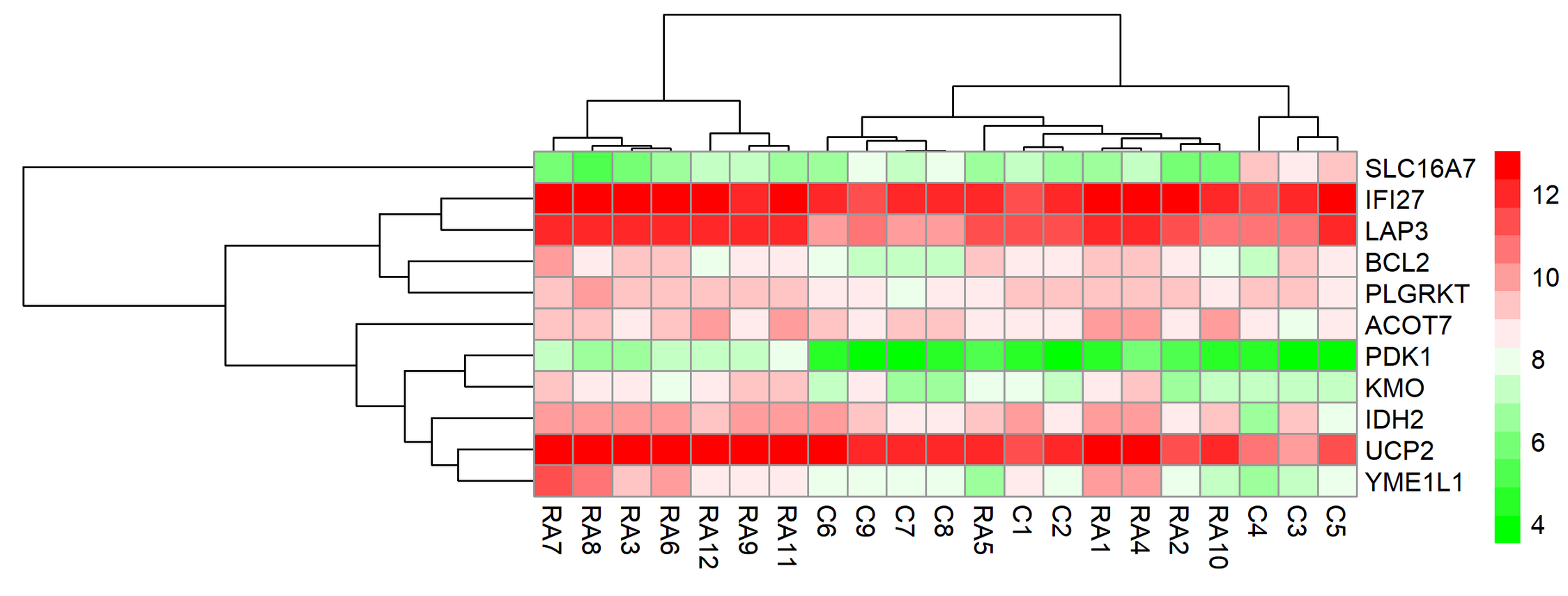

Supplement: S5 Fig — (TIF) [file pone.0224632.s006.tif]

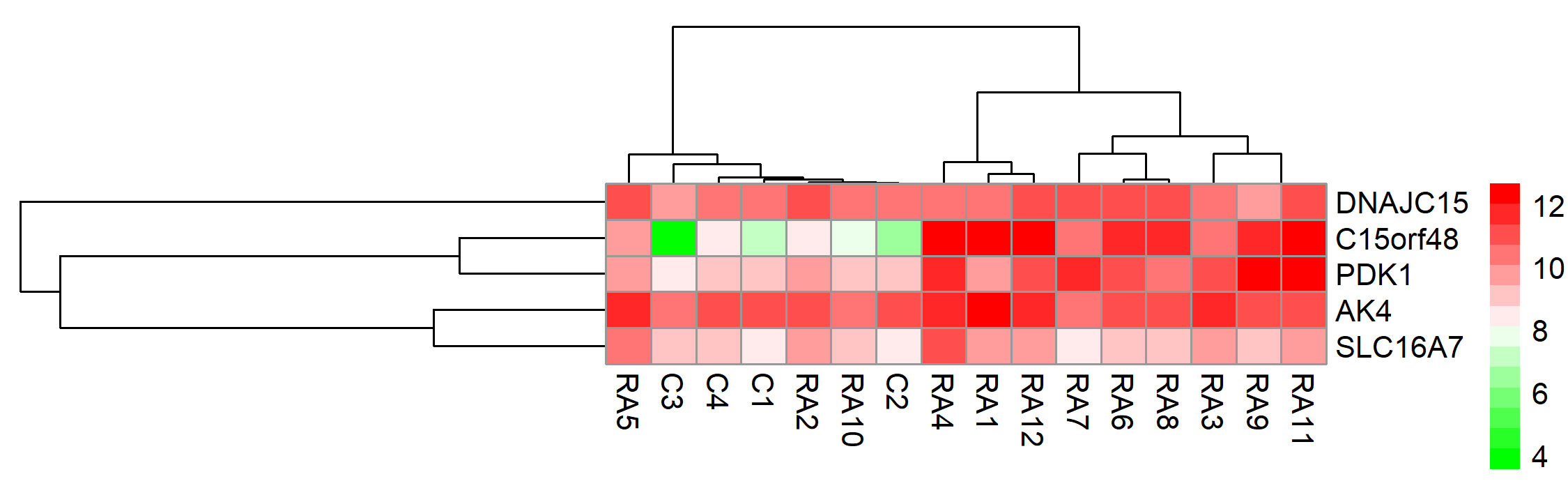

Supplement: S6 Fig — (TIF) [file pone.0224632.s007.tif]

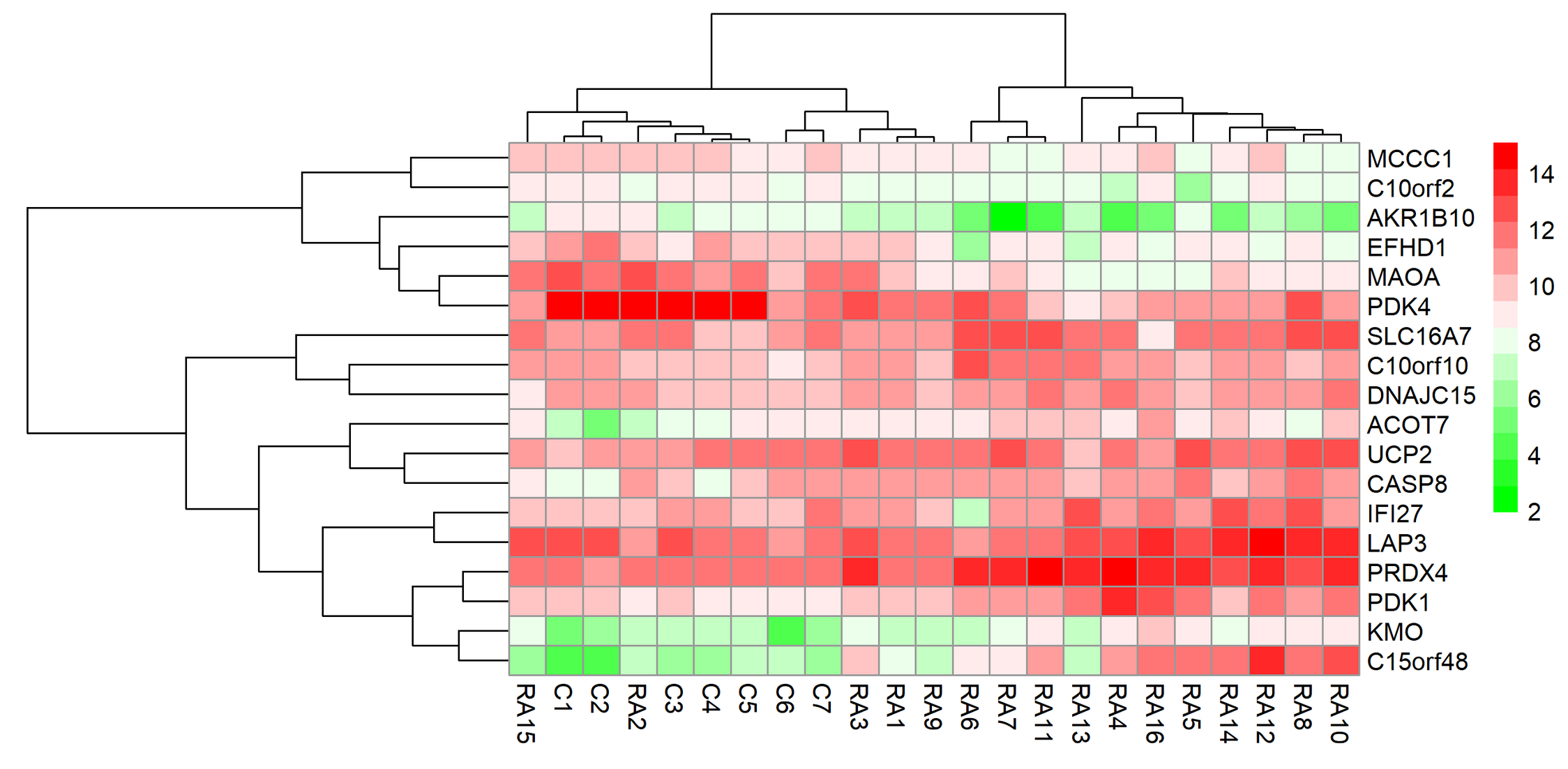

Supplement: S7 Fig — (TIF) [file pone.0224632.s008.tif]

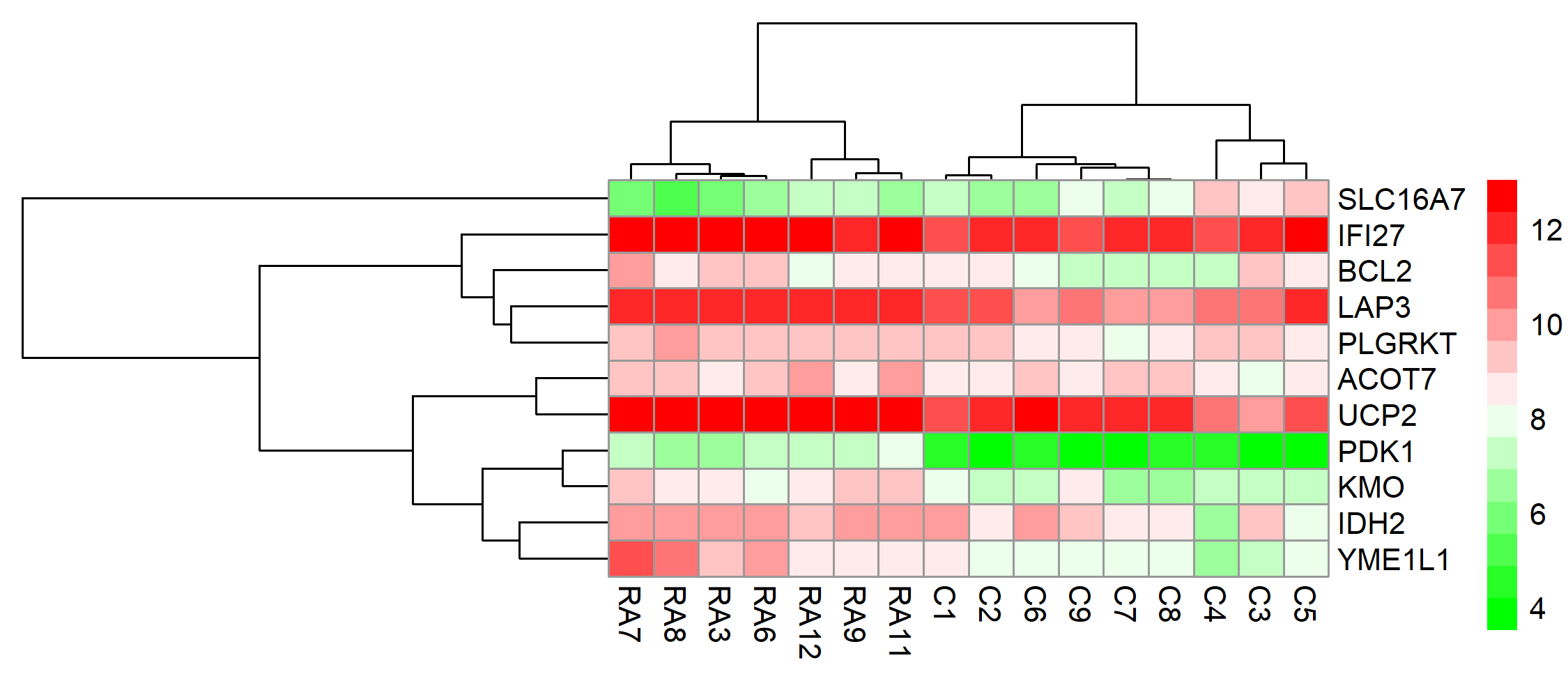

Supplement: S8 Fig — (TIF) [file pone.0224632.s009.tif]

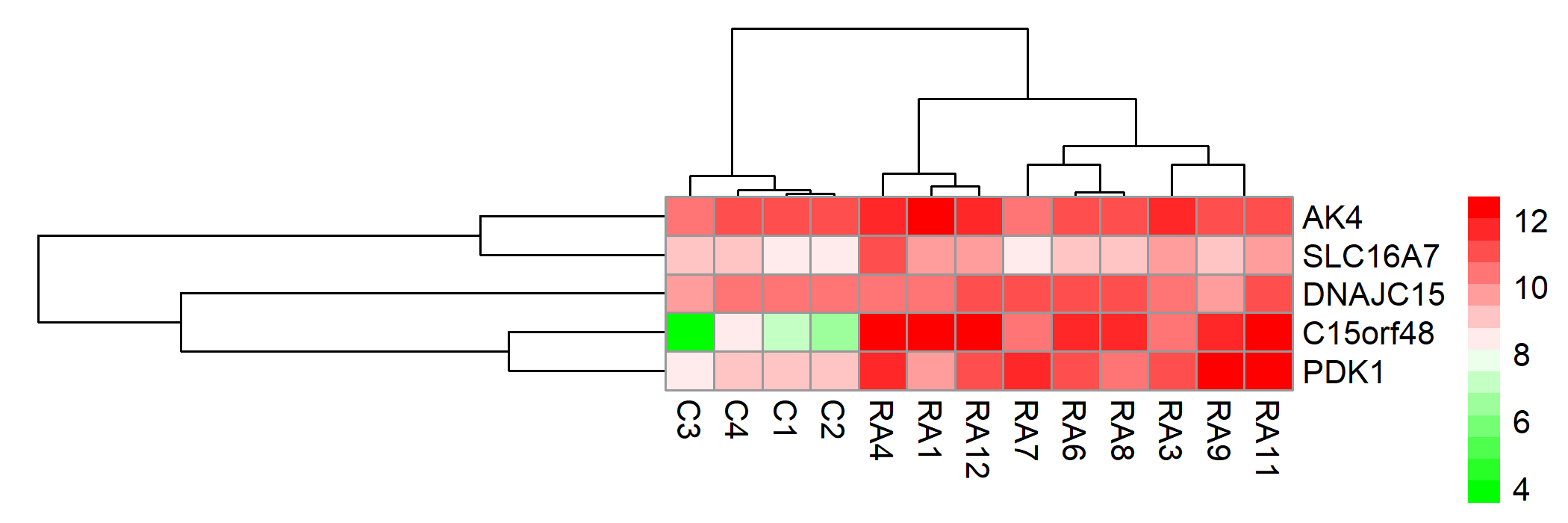

Supplement: S9 Fig — (TIF) [file pone.0224632.s010.tif]

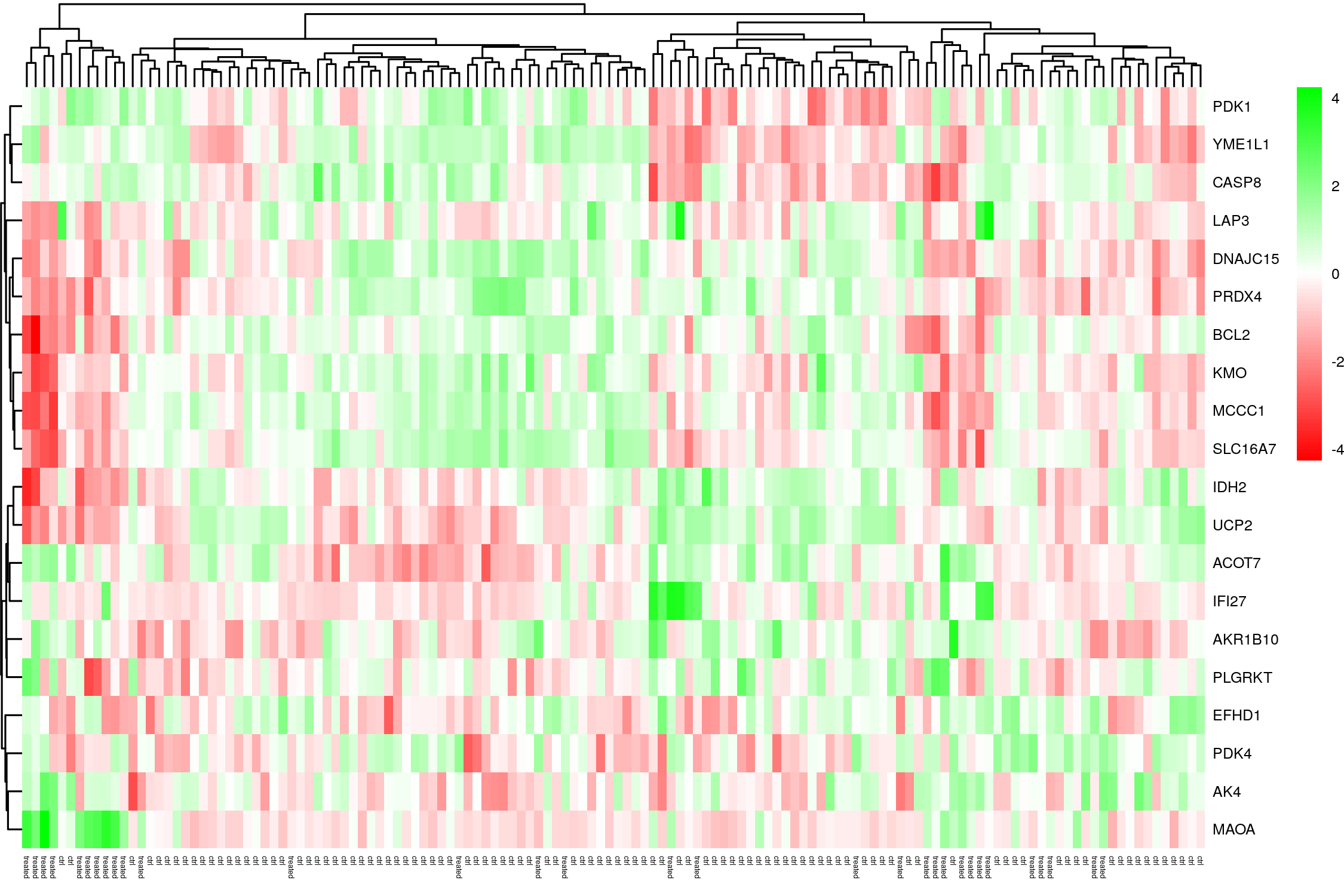

Supplement: S10 Fig — (TIFF) [file pone.0224632.s011.tiff]

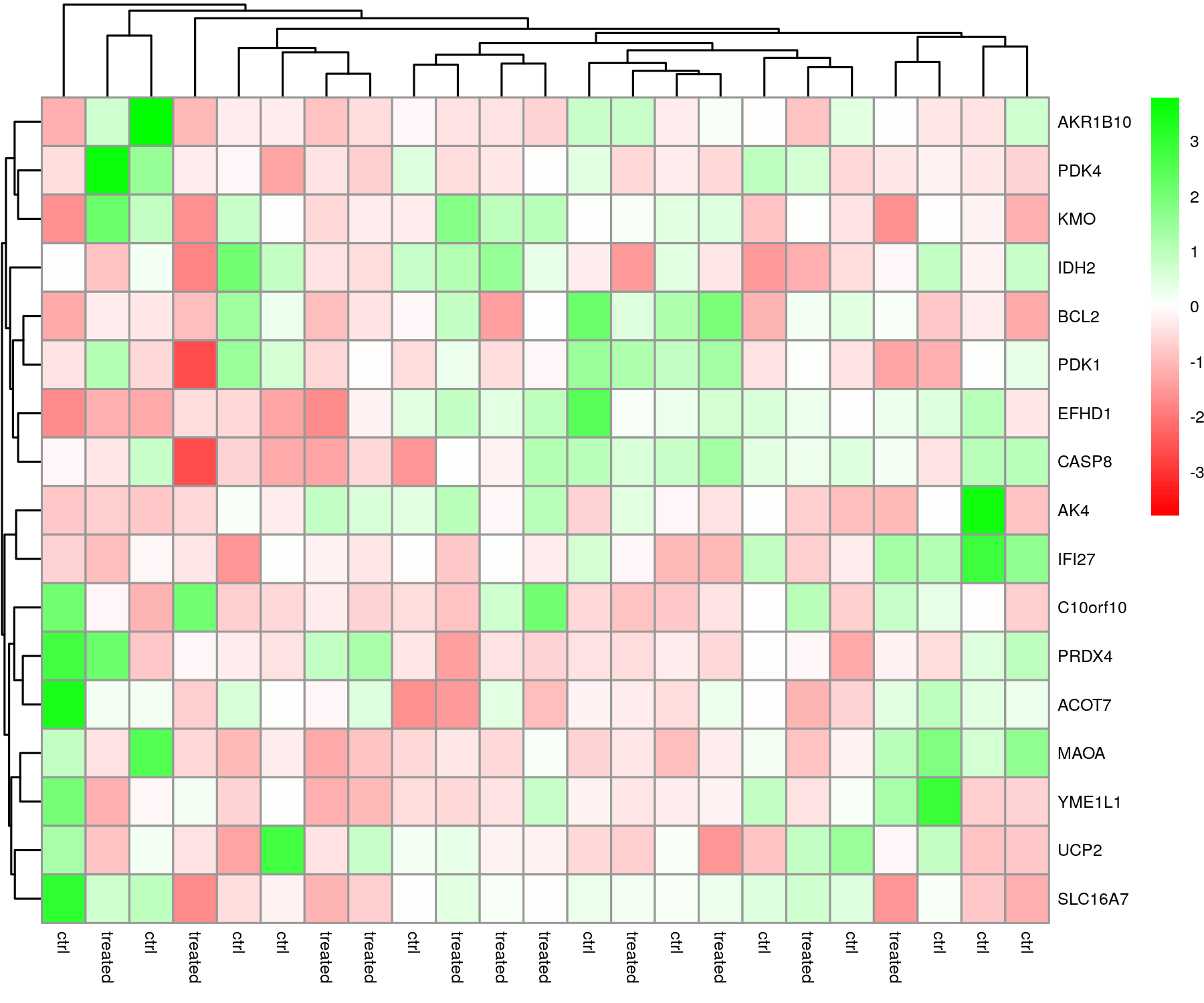

Supplement: S11 Fig — (TIFF) [file pone.0224632.s012.tiff]

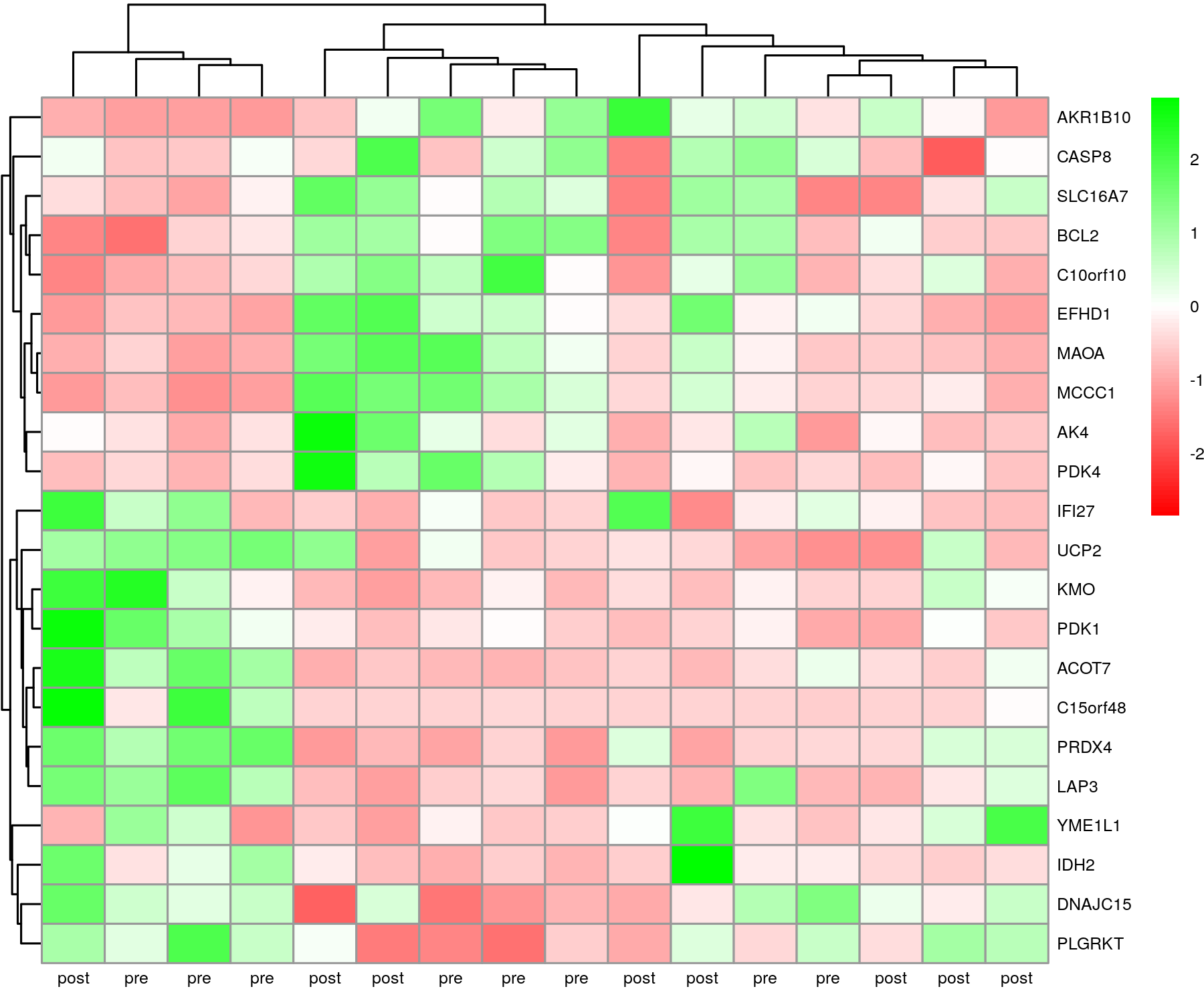

Supplement: S12 Fig — (TIFF) [file pone.0224632.s013.tiff]

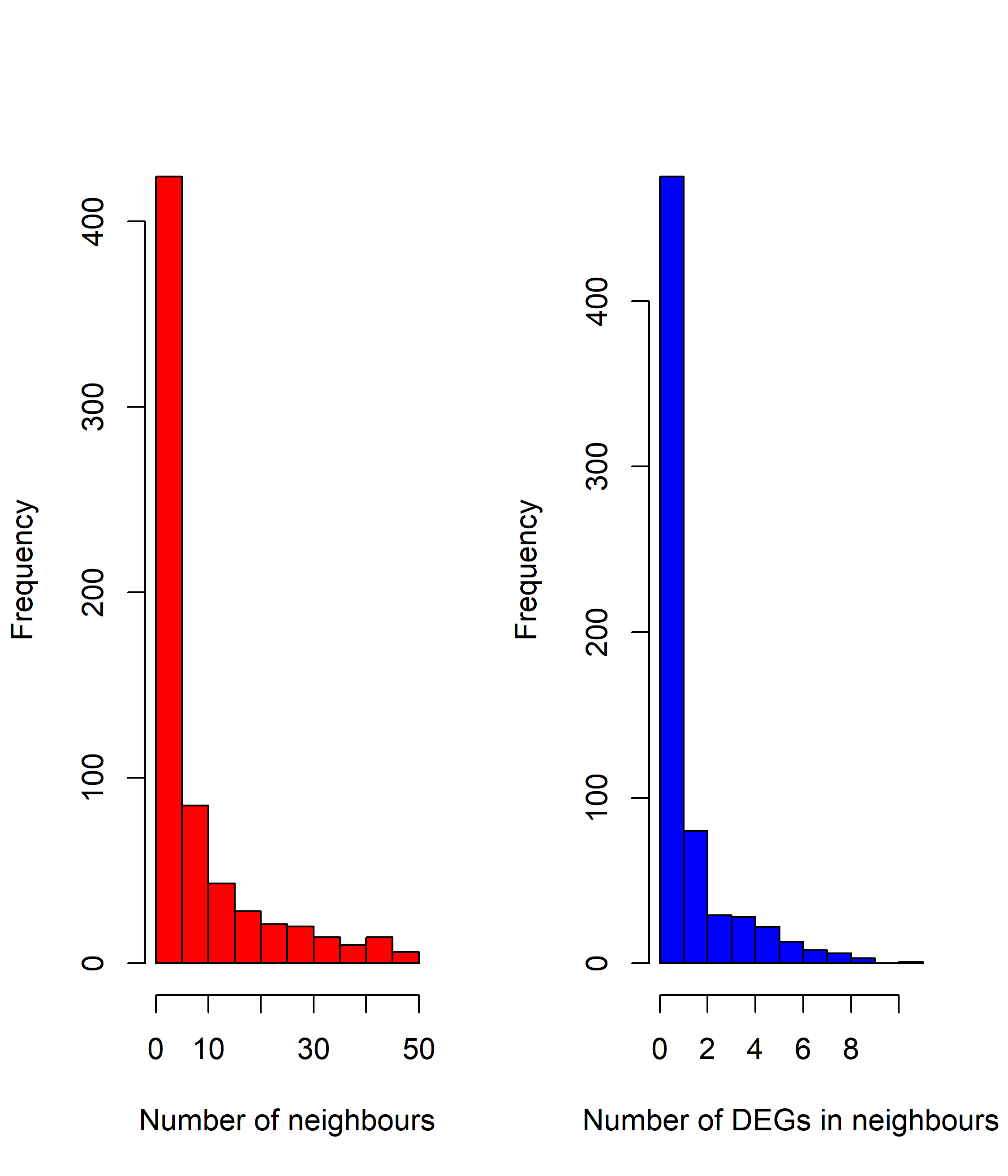

Supplement: S13 Fig — (TIF) [file pone.0224632.s014.tif]

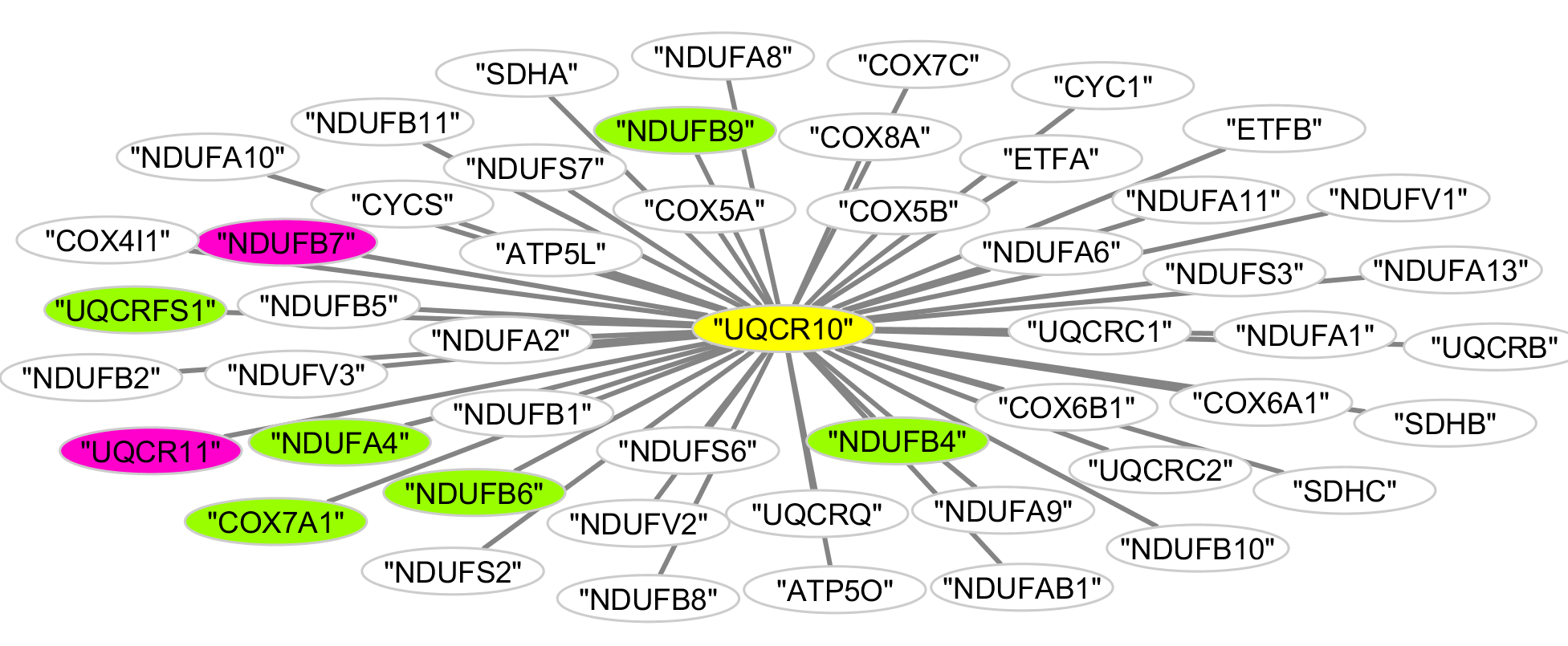

Supplement: S14 Fig — (TIF) [file pone.0224632.s015.tif]

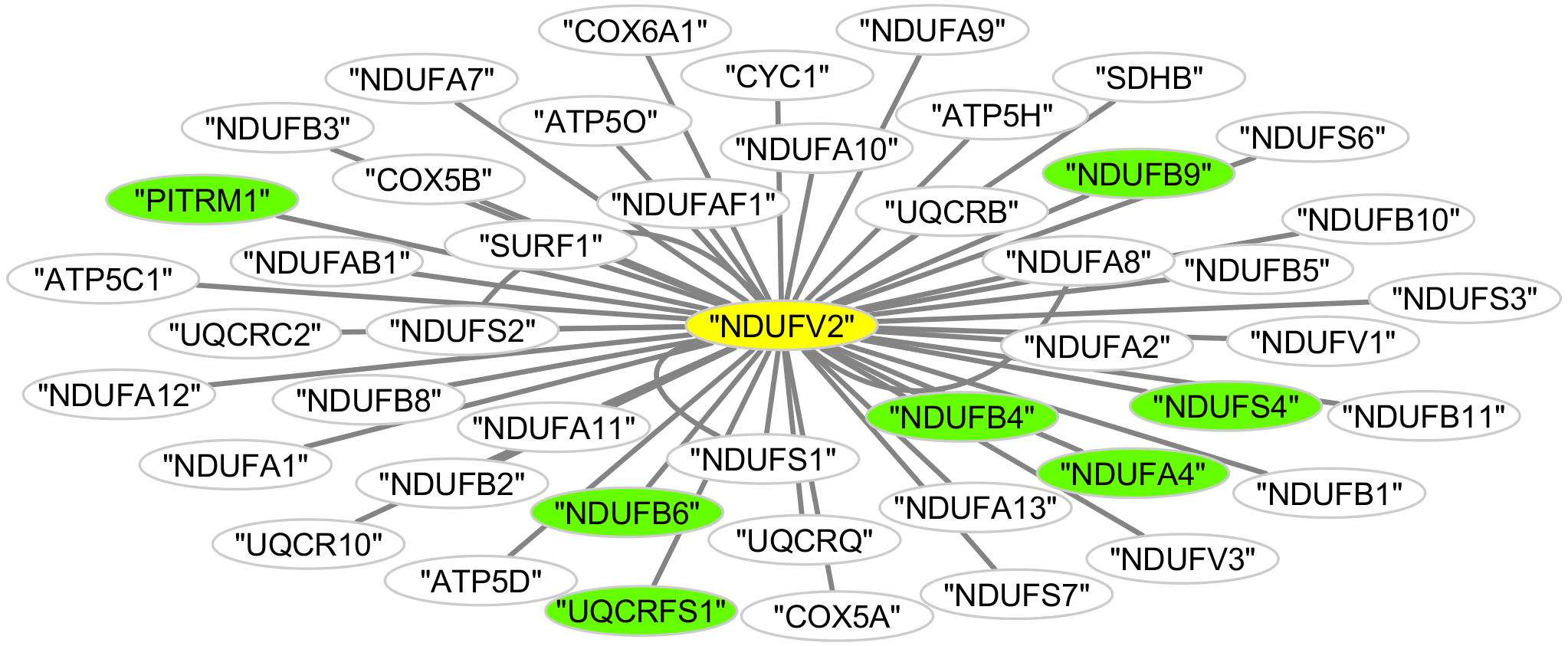

Supplement: S15 Fig — (TIF) [file pone.0224632.s016.tif]

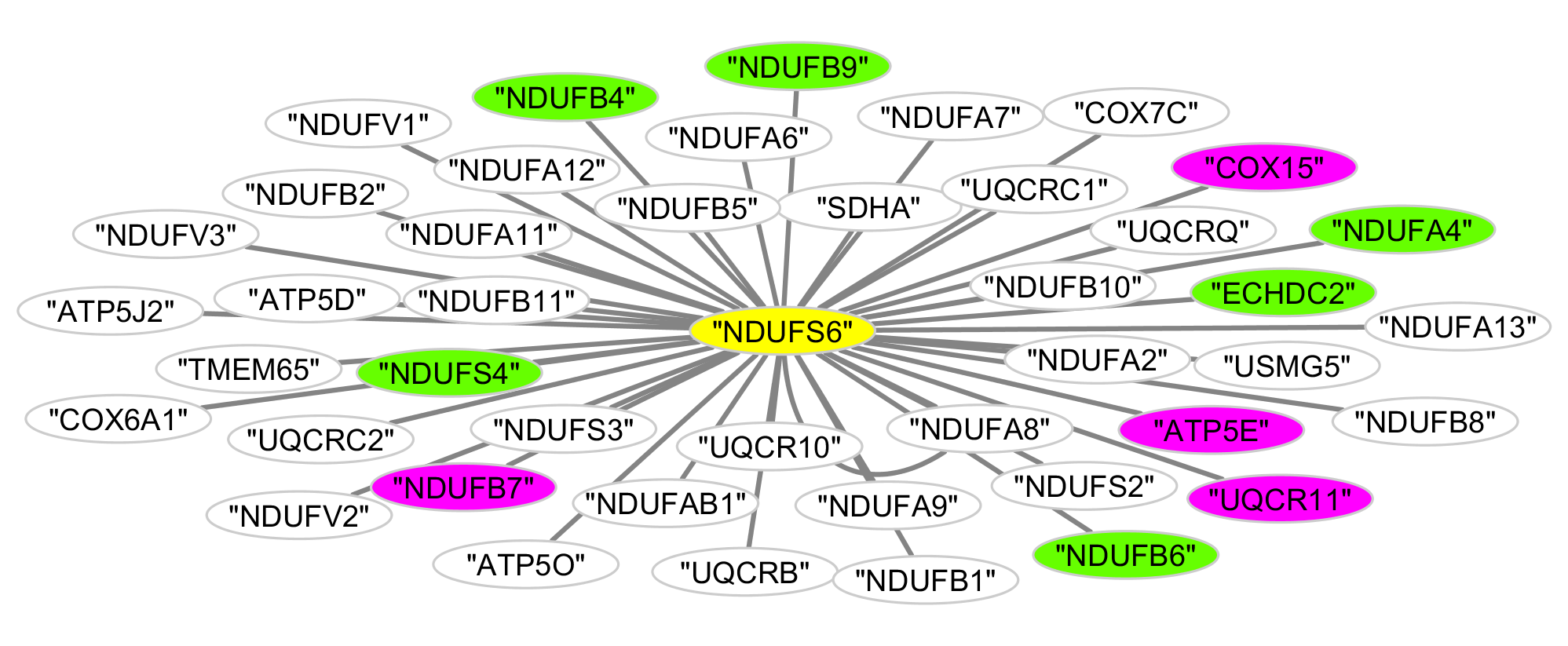

Supplement: S16 Fig — (TIF) [file pone.0224632.s017.tif]

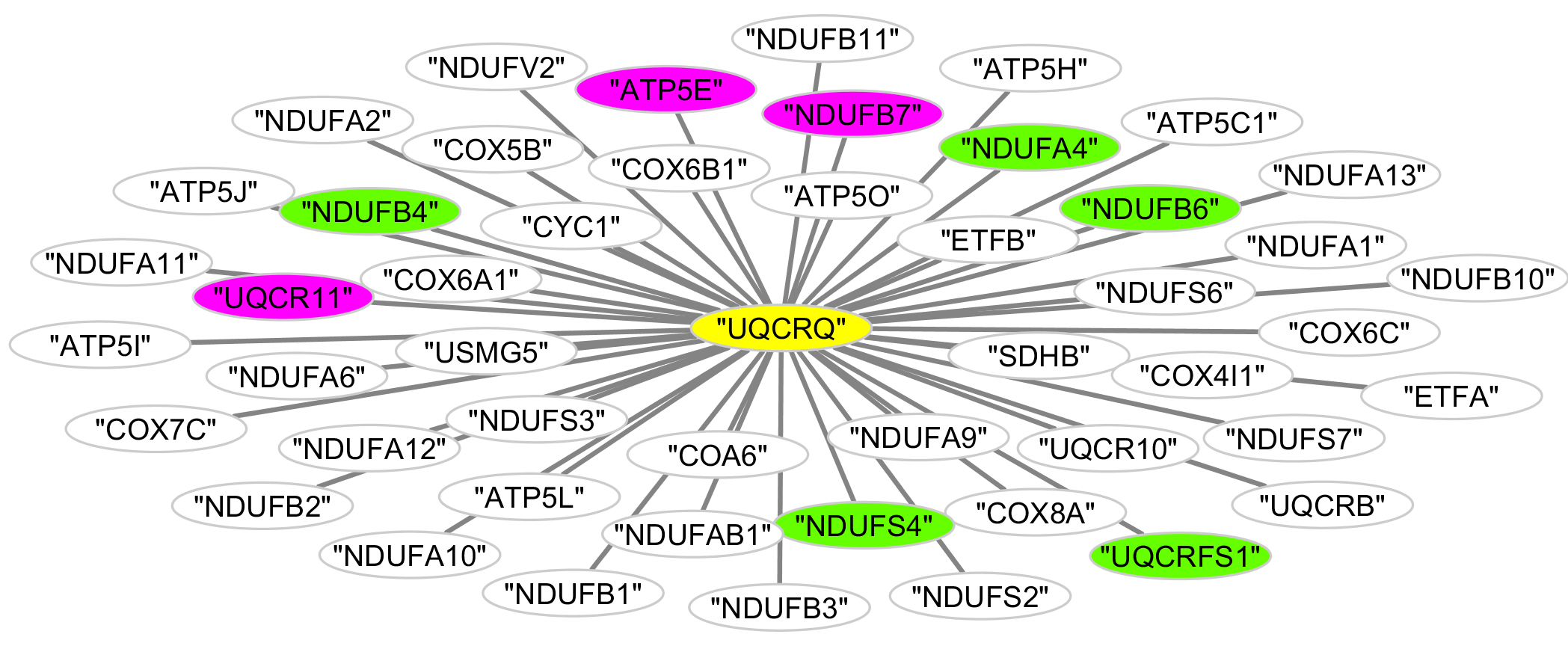

Supplement: S17 Fig — (TIF) [file pone.0224632.s018.tif]
