# Supplementary material for: Mitochondrial dysfunction in rheumatoid arthritis: A comprehensive analysis by integrating gene expression, protein-protein interactions and gene ontology data
Source: PLoS One. 2019 Nov 8;14(11):e0224632. doi: 10.1371/journal.pone.0224632 (PMC6839853; doi:10.1371/journal.pone.0224632)
Supplement: Attachment — Submitted filename: Review.pdf [file pone.0224632.s023.pdf]

The manuscript by Panga and colleagues assesses the mitochondrial dysfunction in Rheumatoid Arthritis (RA). This is an interesting and evolving field exploring the key role of mitochondrial dysfunction in inflammatory disease such as RA. The authors performed a set of integrative analyses of gene expression, protein-protein interactions, and gene ontology from existing data and literature. The data suggested an additional role of nDNA encoded proteins in mitochondrial dysfunction and their relation to inflammation in RA. The authors provided an elegant, important, and very useful tool to analyze nDNA encoded proteins that related to mitochondrial dysfunction in specific disease (in this case, RA).

Major point:

The main concern in this study is that the treatment options for RA as mentioned in Table 3 are known to affect mitochondrial function [1-3]. Therefore, the question becomes how can the authors distinguish between the effects of the disease versus the treatments? The following are two suggested additional experiments that may help further elucidate this concern: (1) test the expression of the same candidate genes in different tissues from patients with non-RA-related diseases that receive the same treatment (if available). (2) test the various treatment effects on mitochondrial function and the expression of the candidate genes (listed on table 2) in an in vitro model such 293T cell lines.

Minor points:

1. Somatic mutations in the mtDNA were found in RA patients [4]. Testing for somatic mutations in the mtDNA and assessment of the expression level of mtDNA encoded genes may provide additional support to your hypothesis.
2. "We also hypothesized a process by which mitochondrial dysfunction could lead to inflammation in RA" is not a new hypothesis [5]

References:


1. Niknahad, H., et al., *Sulfasalazine induces mitochondrial dysfunction and renal injury*. Ren Fail, 2017. **39**(1): p. 745-753.
2. Ryu, J.S., et al., *Prednisolone induces apoptosis in corneal epithelial cells through the intrinsic pathway*. Sci Rep, 2017. **7**(1): p. 4135.
3. Al Maruf, A., et al., *Methotrexate induced mitochondrial injury and cytochrome c release in rat liver hepatocytes*. Drug Chem Toxicol, 2018. **41**(1): p. 51-61.
4. Da Sylva, T.R., et al., *Somatic mutations in the mitochondria of rheumatoid arthritis synoviocytes.* Arthritis Res Ther, 2005. **7**(4): p. R844-51.
5. Fearon, U., et al., *Hypoxia, mitochondrial dysfunction and synovial invasiveness in rheumatoid arthritis.* Nat Rev Rheumatol, 2016. **12**(7): p. 385-97.
